# Assessment of Serum suPAR Levels in Patients with Group 1 and Group 4 Pulmonary Hypertension

**DOI:** 10.3390/jcm14134671

**Published:** 2025-07-02

**Authors:** Abdullah Tunçez, Muhammed Ulvi Yalçın, Hüseyin Tezcan, Bülent Behlül Altunkeser, Bahadır Öztürk, Canan Aydoğan, Aslıhan Toprak, Onur Can Polat, Nazif Aygül, Kenan Demir, Kadri Murat Gürses, Yasin Özen, Fikret Akyürek, Hatice Betül Tunçez

**Affiliations:** 1 Department of Cardiology, Faculty of Medicine, Selçuk University, 42130 Selçuklu-Konya, Turkey; ulviyalcin@gmail.com (M.U.Y.); drhuseyintezcan@hotmail.com (H.T.); bbaltunkeser@hotmail.com (B.B.A.); cano_ylmz@hotmail.com (C.A.); aslihantoprak93@gmail.com (A.T.); onurcanpolat93@gmail.com (O.C.P.); nazifaygul@gmail.com (N.A.); drkenan76@yahoo.com (K.D.); kmuratg@yahoo.com (K.M.G.); ysnozn70@gmail.com (Y.Ö.); 2 Department of Biochemistry, Faculty of Medicine, Selçuk University, 42130 Selçuklu-Konya, Turkey; ozturkbhdr@hotmail.com (B.Ö.); dr.akyurek@hotmail.com (F.A.); hbetltncz@gmail.com (H.B.T.)

**Keywords:** biomarkers, suPAR, pulmonary hypertension, diagnosis

## Abstract

**Background/Objectives:** Pulmonary hypertension (PH) is a progressive disorder with high morbidity and mortality, partly driven by chronic inflammation. Soluble urokinase plasminogen activator receptor (suPAR) reflects immune activation. We evaluated whether suPAR is altered in Group 1 and Group 4 PH and its association with clinical, echocardiographic, and laboratory parameters. **Methods:** We enrolled 44 PH patients (36 in Group 1, 8 in Group 4) and 45 healthy controls. All underwent clinical and echocardiographic assessments; right heart catheterization was performed in the PH patients. Serum suPAR was measured by ELISA. N-terminal pro B-type natriuretic peptide (NT-proBNP) and C-reactive protein (CRP) were also assessed. **Results:** The suPAR plasma levels in the PH group were between 23.91 and 960.8 pg/mL (median: 73.14 p25: 62.77, p75: 167.13). suPAR was significantly higher in PH versus controls (73.14 [62.77–167.13] vs. 65.52 [53.06–80.91] pg/mL; *p* = 0.012). In logistic regression, systolic blood pressure, erythrocyte sedimentation rate, NT-proBNP, and suPAR independently predicted PH. suPAR correlated negatively with six-minute walk distance (r = −0.310) and tricuspid annular plane systolic excursion (r = −0.295) but positively with systolic pulmonary artery pressure (r = 0.241). On multivariate analysis, six-minute walk distance was the only independent correlate of suPAR (*p* = 0.004). suPAR levels did not differ between Group 1 and Group 4 PH. **Conclusions:** suPAR is elevated in Group 1 and Group 4 PH and correlates with functional and echocardiographic indices of disease severity. Larger prospective studies are needed to determine suPAR’s role in diagnosis, risk stratification, and therapeutic decision-making.

## 1. Introduction

Pulmonary hypertension (PH) is an uncommon, progressive, devastating, and severe disease leading to increased morbidity and mortality despite the availability of current treatments [1,2]. The main features of PH are dyspnea, decline of functional capacity, permanent elevation of pulmonary vascular resistance (PVR) and pulmonary arterial pressure (PAP), pulmonary vascular remodeling, and, eventually, loss of right heart functions and death [3].

PH is defined as an increase in mean PAP ≥ 20 mmHg at rest as measured by invasively during right heart catheterization (RHC) [4]. The current guidelines and consensus reports classifies clinically the PH patients into five major groups, namely These are (1) associated, idiopathic, or heritable pulmonary arterial hypertension (PAH) (Group 1 PH); (2) PH secondary to left heart disease (Group 2 PH); (3) PH due to lung disease and/or hypoxia (Group 3 PH); 4) PH secondary to pulmonary artery obstructions, chronic thromboembolic PH or other obstructions (Group 4 PH); and (5) PH with unclear and/or multifactorial mechanisms, hematological disorders, systemic, and metabolic disorders, e.g., (Group 5 PH) [3,4,5]. PAH term is used to describe a subgroup of PH patients (Group 1 PH). Various distinctive hemodynamic measurement criteria must be met for the PAH diagnosis, which are sustained elevation of mean PAP ≥ 20 mmHg, pulmonary artery wedge pressure ≤ 15 mm Hg, and PVR > 2 Wood units (WU), and excluding the other causes of pre-capillary PH, such as PH due to pulmonary diseases (Group 3 PH) and chronic thromboembolic pulmonary hypertension (CTEPH), (Group 4 PH) [4,6].

Experimental animal studies and clinical human studies suggests that chronic inflammation plays a key role in the pathogenesis of PH and in the process of pulmonary vascular remodeling [7,8,9]. Bone-marrow-derived macrophages, dendritic cells, and lymphocytes were shown histologically around the remodeling vessels in rats with monocrotaline-induced PH [10,11]. Various serum pro-inflammatory cytokine levels (IL [interleukin] 1β, IL6, MCP [monocyte chemoattractant protein]-1, and TNF [tumor necrosis factor]-α) were elevated and are linked to poor prognosis in patients with PAH [12].

The urokinase type plasminogen activator receptor (uPAR) is a three-homologous-domain (D1-D2-D3) GPI (glycosylphosphatidylinositol)-anchored cell membrane receptor that converts inactive plasminogen into active plasmin, mainly expressed on immune active cells, activated T cells, macrophages, and vascular endothelial cells [13,14]. The soluble urokinase plasminogen activator receptor (suPAR) is the circulating soluble form of the uPAR, generated after the proteolytic cleavage of membrane-linked uPAR; it can be detected in plasma, urine, and cerebrospinal fluid. Increased serum concentrations of suPAR reflects the level of immune activation and inflammatory status in various pathological conditions ranging from bacterial and viral infectious diseases, cancers, and heart failure to chronic inflammatory disorders and chronic renal and chronic liver diseases [15,16,17,18,19,20,21].

Elevated levels of suPAR are associated with the presence and severity of coronary artery disease, coronary artery calcifications and microvascular dysfunction in patients with non-obstructive coronary artery disease [22,23,24,25]. Elevated levels of suPAR also predict mortality in patients with heart failure, asymptomatic aortic stenosis, ST-segment elevation myocardial infarction undergoing primary percutaneous intervention, systemic inflammatory response syndrome, and staphylococcus bacteremia [26,27,28,29].

Various circulating pro-inflammatory biomarkers are elevated in patients with various subgroups of PH [30,31,32]. To the best of our knowledge, there is limited data in the literature evaluating the levels of suPAR in patients with Group 1 and Group 4 PH, and in this study, we aimed to evaluate whether Group 1 and Group 4 PH was accompanied by a change in the serum levels of suPAR and if there was a considerable difference in the suPAR levels amongst in patients with Group 1 and Group 4 PH. Furthermore, we aimed to investigate the association between suPAR and N-terminal pro B-type natriuretic peptide (NT-proBNP), C-reactive protein (CRP), functional status, 6 min walking distance (6MWD) other baseline clinical and echocardiographic characteristics.

## 2. Materials and Methods

### 2.1. Patient Population

In this single-center, prospective, observational study, we enrolled consecutive patients who were followed by our PH clinics at the Selçuk University Faculty of Medicine, with the diagnosis of Group 1 PH (iPAH, congenital heart disease associated PH, connective tissue disease (CTD)-associated PH, heritable PAH, etc.) and Group 4 PH (CTEPH). All patients provided written informed consent. The protocol of the study was approved by the local institutional Ethics Committee.

Group 1 and Group 4 PH were defined by the met of the following hemodynamic criteria obtained during RHC: mean PAP ≥ 20 mmHg at rest, PCWP ≤ 15 mmHg, and PVR > 2 Woods Unit (WU) [4,5]. Both ventilation-perfusion scintigraphy (V/Q scan) and computed tomography pulmonary angiography (CTPA) were applied for the diagnosis of the CTEPH (Group 4 PH) [3,33]. The control group consisted of age- and sex-matched healthy individuals with low probability of PH [4]. Patients with right ventricular/left ventricular (RV/LV) basal diameter/area ratio < 1.0, early diastolic pulmonary regurgitation velocity < 2.2 m/s, right atrial area < 18 cm^2^, pulmonary artery diameter < 25 mm, inferior vena cava diameter < 21 mm with normal inspiratory collapse, tricuspit regurgitation (TR) jet velocity < 2.8 m/s, and estimated systolic PAP < 30 mmHg measured by TR jet velocity on transthoracic echocardiography (TTE) were defined as low probability of PH and were included in the control group [4]. All TTE studies were performed with Vivid E9 echocardiography system (Vivid E9, GE Vingmed, Horten, Norway).

Patients with Groups 2, 3, and 5 PH, vasoreactive PH, cardiogenic shock, serum creatinine > 2.5 mg per deciliter, acute or chronic glomerulonephritis, active infection or sepsis, blood transfusion within 3 months, active chronic inflammatory and rheumatic diseases, known malignancy, presence of obstructive hepatobiliary disease, cirrhosis and chronic liver disease, and acute coronary syndromes, as well as those who refused to provide written informed consent, were excluded from the study.

Baseline clinical characteristics and co-morbidities of the patients with PH and control group were collected from medical records and electronic database of our center, including hypertension, diabetes mellitus (DM), cerebrovascular and cardiovascular diseases, among other comorbidities. Functional capacity, 6MWD, symptom status (angina pectoris, exertional or rest dyspnea, syncope, etc.), biochemistry parameters, and complete blood count (CBC) were also assessed for both groups. The electrocardiographies (ECG) of all patients included in this study were evaluated. The TTE reports of all patients were reviewed and the tricuspid annulus peak systolic excursion (TAPSE), pericardial effusion, LV ejection fraction (LVEF), and systolic, diastolic, and mean PAP and right atrial area (RAA) measurements were recorded. The targeted/specific medical therapies of patients were classified as endothelin receptor antagonists (ERAs), phosphodiesterase type 5 inhibitors (PDE-5is), prostacyclin analogs, and soluble guanylate cyclase stimulators. Patients on combination therapies were also defined.

Hemodynamic evaluation was performed by RHC using the right or left femoral veins. Left heart catheterization was also performed to all PH patients to measure the left ventricular end diastolic pressure by using the right femoral artery. Cardiac output (CO) was calculated by Fick’s method. Acute vasoreactivity test was performed for all patients with Group 1 PH after baseline RHC. Although the current guidelines do not recommend acute vasoreactivity testing for all patients considered to have Group 1 PH, acute vasoreactivity testing was performed for all patients considered to have Group 1 PH due to the reimbursement rules of the state health insurance. The protocol of the acute vasoreactivity test with adenosine had been previously described by the European Society of Cardiology’s (ESC) guidelines for diagnosis and treatment of pulmonary hypertension [3]. Infusion of adenosine was given by intravenous route. Adenosine infusion started at 50 mcg/kg/min. If the patient tolerated, the adenosine infusion rate was increased by 50 mcg every two minutes until reaching the target dose of 350 mcg/kg/min. If side effects of adenosine occurred, the infusion was stopped. A positive acute vasoreactive response is defined as a reduction in the mean PAP ≥ 10 mmHg to reach an absolute value of mean PAP ≤ 40 mmHg with an increased or unchanged CO [3]. The findings of vasoreactivity tests were recorded.

Blood samples were taken from each subject after 12 h fasting by a cubital venipuncture avoiding venous stasis to an evacuated serum separator tube. The samples were centrifuged at 3000 rpm for 10 min within 1 h after collection. After centrifugation, the serum samples were transferred to Eppendorf tubes and stored at −80 °C until assayed. The biochemistry parameters were measured by a chemistry autoanalyzer (ARCHITECT c16000, Abbott Diagnostics, Abbott Park, IL, USA) via enzymatic colorimetric methods. The EDTA tubes were used for hematological tests. The CBC parameters were analyzed by an autoanalyzer (Coulter Gen-S Hematology Analyzer, Beckman Coulter Corp, Hialeah, FL, USA) within 5 min of blood sampling. Serum human suPAR levels (Catolog no: E3759Hu, Bioassay Technology Laboratory, Shanghai, China) were determined by using the enzyme-bound immunosorbent test technique with sandwich ELISA, as suggested by the supplier. The serum concentration of the NT-proBNP was measured using the chemiluminescence immunometric method of the IMMULITE 2000 autoanalyzer (Siemens, Germany).

### 2.2. Statistical Analysis

Statistical analyses were performed using the Statistical Package for Social Sciences software (IBM SPSS Statistics for Windows, version 21.0; IBM Corp., Armonk, NY, USA). The normally distributed parameters were presented as mean ± standard deviation and the skewed parameters were expressed as median (interquartile range: 25–75%). Categorical variables were presented as frequencies and percentages (%). The Kolmogorov–Smirnov test was used to test the normality of distribution. The Mann–Whitney U test or Student’s *t*-test were used to compare continuous variables where appropriate. The categorical variables were compared using Fisher exact or Chi-square tests where appropriate. To determine the independent associates of the presence of PH, binomial logistic regression analysis was performed. Spearman or Pearson correlation analyses were used to investigate the factors related to suPAR levels. Linear regression analysis was performed to determine the independent associates of suPAR levels. Possible confounding factors were tested in the univariate regression model and confounders with a *p* value of <0.2 included in the backward method of the multivariate regression model. *p* < 0.05 was considered statistically significant for all tests.

## 3. Results

The baseline clinical characteristics of the groups are presented in Table 1. There were no significant differences between the two groups in terms of age, gender, body mass index (BMI), and smoking status. The groups were also comparable regarding baseline white blood cell (WBC) count, glomerular filtration rate (GFR), and CRP levels (Table 1).

### 3.1. Clinical, Laboratory, and Echocardiographic Parameters

**Figure 1:**
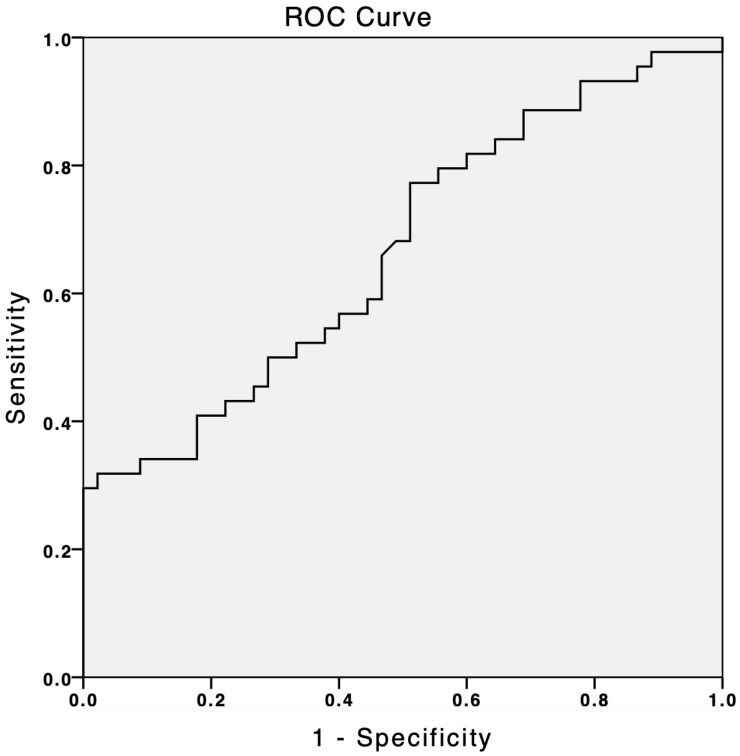
Receiver operating aharacteristic analysis of suPAR levels in predicting the PH presence.

In total, we enrolled 89 patients in this study, 44 of whom were included in the group of PH and 45 in the control group. Of the 44 patients included in the PH group, 36 patients (82%) were in Group 1 PH and 8 patients (18%) were in Group 4 PH (Table 1). Of the 36 patients in Group 1 PH, 22 patients were iPAH, 12 were congenital heart disease-associated PAH, and 2 were connective tissue disease-associated PAH (Figure 1). The mean age of patients in the group of PH was 51.05 ± 18.18 years and 27.3% were male. The mean age of patients in the control group was 51.24 ± 12.48 years and 26.7% were male (Table 1). The baseline demographic, clinical, laboratory, and echocardiographic parameters of both groups are listed in Table 1. Among the clinical parameters, systolic blood pressure (110 [100–130] mmHg in the PH group and 120 [100–130] mmHg in the control group, *p* = 0.041), heart rate (82.50 [70–95] beats per minute in the PH group and 70 [70–80] beats per minute in the control group, *p* = 0.005) and 6-MWD (372.50 [312.50–407.50] m in PH group and 650 [622.50–680] m in the control group, *p* < 0.001) were significantly different in patients with PH when compared to controls (Table 1). Among the laboratory parameters, levels of hemoglobin (12.65 ± 2.33 g/dL in the PH group and 14.54 ± 1.41 g/dL in the control group, *p* < 0.001) and NT- proBNP (442.00 [120.75–1516.75] pg/mL in the PH group and 37.60 [24.40–80.50] pg/mL in the control group, *p* < 0.001) were significantly different between both treatment groups (Table 1). The baseline TTE findings are shown in Table 1. All echocardiographic parameters were significantly different between the two groups (Table 1). The other parameters, including BMI, smoking history, diabetes mellitus, and WBC count, were similar between the two groups (Table 1).

### 3.2. suPAR

The suPAR plasma levels in the PH group were between 23.91 and 960.8 pg/mL (median: 73.14 p25: 62.77, p75: 167.13). The suPAR levels were found to be significantly elevated in patients with PH compared to the controls (73.14 [62.77–167.13] pg/mL in PH group and 65.52 [53.06–80.91] pg/mL in the control group, respectively, *p* = 0.012) (Table 1). Binary logistic regression analyses were performed to define the independent predictors of the presence of the PH. Univariate regression analyses revealed that systolic blood pressure, heart rate, hemoglobin, erythrocyte sedimentation rate, NT-proBNP, and suPAR levels were significantly associated with the presence of PH (Table 2). Multiple regression analyses showed that systolic blood pressure (OR = 0.875 [0.780–0.981], *p* = 0.022), erythrocyte sedimentation rate (1.177 [1.038–1.335], *p* = 0.011), NT-proBNP (OR = 1.025 [1.009–1.042], *p* = 0.003), and suPAR (OR: 1.047 [1.004–1.093], *p* = 0.032) levels were also independent predictors for the presence of PH (Table 2). A subgroup comparison of clinical and hemodynamic parameters between Group 1 and Group 4 PH patients is provided in Table 3. Our study identified a suPAR cut-off value of 61.8 pg/mL, which demonstrated a sensitivity of 77% and a specificity of 50% for predicting the presence of PH. This prediction had an area under the curve (AUC) of 0.666 (95% CI 0.554–0.779, *p* < 0.007) (Figure 1).

We performed correlation analyses to evaluate the relationship between the suPAR levels and the baseline clinical, laboratory, and echocardiographic parameters of the study population (Table 4). 6-MWD (r = −0.310, *p* = 0.001) and TAPSE (r = −0.295, *p* = 0.005) were found to be negatively correlated with the suPAR levels, and systolic PAP (r = 0.241, *p* = 0.023) was positively correlated with the suPAR levels (Table 4). Lineer regression analyses were performed to determine the independent associates of the suPAR levels in the study population (Table 5). Univariate lineer regression analyses revealed that heart rate (*p* = 0.044), 6-MWD (*p* = 0.004), RAA (*p* = 0.013), and TAPSE (*p* = 0.013) were found to be independently associated with the suPAR levels (Table 5). Multiple lineer regression analyses showed that only the 6-MWD was independently associated with the suPAR levels (*p* = 0.004) (Table 5).

### 3.3. Comparison of Patients with Group 1 and Group 4 PH

The demographic, clinical, laboratory, echocardiographic, and heart catheterization parameters of the study population regarding the type of PH are shown in Table 3. A total of 36 patients were in Group 1 PH, and 8 patients were in Group 4 PH. The demographic and clinical parameters were similar regarding the type of PH, except 6-MWD. 6-MWD was higher in patients with Group 4 PH than in patients with Group 1 PH (437.50 ± 68.19 m in patients with Group 4 PH and 345.14 ± 71.07 m in patients with Group 1 PH, respectively, *p* = 0.002). Among the laboratory parameters, only the WBC count levels (8.05 ± 3.00 × 10^9^/L in patients with Group 4 PH and 5.55 ± 1.19 × 10^9^/L in patients with Group 1 PH, *p* = 0.027) were significantly higher in patients with Group 1 PH than in patients with Group 4 PH (Table 3). The suPAR levels were similar between both PH groups, and there was no statistically significant difference between the two PH groups (70.57 (63.90–235.76): pg/mL in patients with Group 1 PH and 75.48 (62.31–80.08) pg/mL in patients with Group 4 PH (*p* = 0.800) (Table 3). The TTE and RHC parameters were similar between Group 1 and Group 4 PHs (Table 3). PVR was higher in patients with Group 1 PH than in patients with Group 4 PH, but this difference could not reach the statistically significant level (7.62 ± 2.57 woods in patients with Group 1 PH and 5.43 ± 4.29 woods in patients Group 4 PH, respectively, *p* = 0.063). Also, the specific/targeted medications of our study population are presented in Table 3 according to type of PH group. In the Group 1 PH, 38.9% of the patients were on a single PAH-specific drug treatment, 44.4% on a double-combination PAH-specific drug treatment, and 16.7% on triple-combination PAH-specific medications. On the other hand, all patients in the Group 4 PH were on single-specific drug treatment (riociguat).

## 4. Discussion

The present study demonstrated that suPAR levels significantly elevated in patients with Group 1 and Group 4 PH compared to healthy controls. Furthermore, suPAR was identified as an independent predictor of PH’s presence, alongside established markers such as NT-proBNP. To the best of our knowledge, this is the first study to specifically investigate the relationship between suPAR levels and PH, particularly in Group 1 and Group 4 PH, and to explore its potential as a biomarker in this context.

PH is a heterogeneous, chronic, progressive, and malignant pulmonary vascular disease characterized by angioneogenesis, vascular smooth muscle cell proliferation, vasoconstriction, inflammation, extracelluar matrix remodeling, fibrosis, and thrombosis as a result of imbalance between vasoconstrictor, vasodilatory, and inflammatory mediators [32,34,35,36]. These progressive and uncontrolled pathophysiological changes results in increased PVR, leading to elevated PAP and ultimately right ventricular failure and death [32,37]. PAH is subgroup of PH and classified as Group 1 PH in the current guidelines and consensus reports [4]. Although the real numbers of the incidence and the prevalence of PAH are not clear due to rare nature of disease, previous registries demonstrated that the incidence of PAH is between 2.0 and 2.4 cases per million and the prevalence of PAH is between 10.6 and 15 cases per million [38,39]. Despite the current advances in the medical treatment options of PAH in the last 30 years, PAH remains associated with poor survival [2,40,41]. Hooper MM et al. analyzed the data of the newly diagnosed PAH patients enrolled into the Comparative, Prospective Registry of Newly Initiated Therapies for Pulmonary Hypertension (COMPERA) registry [42]. In this study, Hooper et al. demonstrated that patients in the high-risk cohort showed high mortality rates (mortality rate %20.2, n = 276) 1 year after diagnosis [42]. In a similar trial, with the Swedish PAH registry, Kylhammar et al. demonstrated that patients in the high-risk cohort showed high mortality rates at 1 year (%30) [43].

Large-scale, multicenter, nationwide registry studies showed that the majority of the patients with PAH were at NYHA functional class 2 and class 3 and again the majority of the patients with PAH were at intermediate 1-year mortality risk status according to the current guidelines [3,42,44]. A recent Greek registry study by Arvanitaki et al., which included 231 patients with PAH, demonstrated some results supporting this situation and showed that more than 50% of patients with PAH were in the World Health Organization’s (WHO) functional class 2 and more than 30% of patients with PAH were in the WHO’s functional class 3 at baseline [45]. Moreover, this registry by Arvanitaki et al. demonstrated that 57% of patients with PAH were at intermediate (5–10%) 1-year mortality risk status at baseline [45].

For the reasons mentioned above, in recent years, researchers focused intensely on new, non-invasive, reproducible, applicable, and easily available modalities (e.g., imaging, biomarkers) to further improve the time of first diagnosis, facilitating clinical management decisions, and to further strengthen the predictive power of the risk assessment tools developed for the patients with pulmonary hypertension. In our study, we showed that suPAR is independently associated with the PH presence in patients with Group 1 and Group 4 PHs.

suPAR is the circulating soluble form of the UPAR, generated after the cellular shedding of a section of uPAR. suPAR is an inflammatory biomarker, reflecting low-grade inflammatory status. It is mainly secreted from endothelial cells or inflammatory cells. suPAR levels are elevated mainly during the inflammatory processes [46]. There is limited data in the current literature about the association between suPAR levels, PAH, and other forms of PH.

Our results show that suPAR levels were significantly higher in the PH patients compared to controls (73.14 [62.77–167.13] pg/mL vs. 65.52 [53.06–80.91] pg/mL, *p* = 0.012, Table 1). This finding aligns with the growing body of evidence that implicates inflammation in the pathogenesis of PH. suPAR, a marker of immune activation and chronic low-grade inflammation, is known to be elevated in various cardiovascular and inflammatory conditions [46,47]. In PH, inflammation plays a critical role in vascular remodeling, endothelial dysfunction, and disease progression. The elevated suPAR levels observed in our study may reflect the underlying inflammatory processes driving PH pathophysiology.

Chronic inflammation is a hallmark of PH, and elevated levels of inflammatory markers such as IL-6, TNF-α, and CRP have been observed in PH patients. [7,8]. While our study did not measure these cytokines, the elevated suPAR levels observed in our cohort suggest that suPAR may serve as a surrogate marker of systemic inflammation in PH.

Levi et al. performed an important animal study investigating the possible role of the plasminogen system in the pathogenesis of the PH [48]. In their study, mice with experimentally deficient of the gene encoding the uPAR (uPAR −/−) were exposed to hypoxic conditions. They observed that uPAR deficiency, as a result of the absence of the uPAR gene (−/−), protects mice against the development of hypoxia-induced PH and pulmonary arterial remodeling [48].

Extracellular matrix (ECM) remodeling plays a significant role in the pathogenesis of PH [34]. Endothelial dysfunction, pulmonary arterial smooth muscle dysfunction, excessive inflammatory status, and increased levels of pro-inflammatory cytokines result in an imbalance between proteolytic enzymes and the tissue inhibitor of matrix metalloproteinases. This imbalance between proteolytic enzymes and the tissue inhibitor of matrix metalloproteinases results in ECM remodeling. ECM remodeling and pulmonary vascular stiffness occur at the early stages of PH. ECM remodeling often occurs with the increased collagen storage, cross-linkage of collagen, and destruction of elastic laminae [34]. Previous studies showed that suPAR levels successfully reflect the low-grade activation of the immune system [46,47]. At the same time, suPAR coordinates cellular adhesion, cellular migration, cellular proliferation, and ECM remodeling [49,50]. Several prior studies demonstrated the association between suPAR levels, ECM remodeling, and ECM biomarkers. Kruger et al. observed that an extracellular matrix biomarker, Fibulin-1, is independently and closely related to NT-proBNP and suPAR levels in patients with aortic valve stenosis [51]. They showed that increased levels of Fibulin-1 were independently associated with the increased levels of NT-proBNP and suPAR [51]. In parallel with these results, du Plooy et al. showed that serum Fibulin-1 levels independently associated with serum suPAR levels in African and Caucasian men [52]. Du Plooy et al. concluded that subclinical inflammation within the ECM of the endothelial tissue, reflected by the suPAR levels, may promote the development of cardiovascular fibrosis and sclerosis [52].

Bai et al. demonstrated the importance of the extracellular matrix remodeling in the pathogenesis of the PH [53]. They showed that legumain, a cysteine proteinase expressed mainly in macrophages, promotes the development of PAH by activating the matrix metalloproteinase (MMP)-2/Transforming Growth Factor (TGF)—β_1_-signaling pathway [53]. The authors showed that inhibition of legumain in mice markedly ameliorated the development of experimental PAH, and they suggested that the inhibition of legumain may be effective for the treatment of PAH in the future [53].

ECM remodeling has an important role in the pathogenesis of systemic sclerosis (SSc). SSc is a connective tissue disease characterized by fibrosis, vascular dysfunction, and immune dysregulation. It is often associated with PH, particularly Group 1 PH (pulmonary arterial hypertension, PAH). Legány et al. conducted a study comparing patients with SSc and healthy subjects in terms of suPAR levels [54]. They showed that suPAR levels are elevated in patients with SSc [54]. They also showed that suPAR levels were correlated with the parameters of pulmonary fibrosis and the presence of digital ulcers, Raynaud’s phenomenon, and nailfold capillaroscopic abnormalities, which are all the results of ECM remodeling and microvascular abnormalities [54]. Butt et al. also showed that suPAR levels are strongly associated with pulmonary involvement in patients with SSc, but suPAR is not associated with PAH presence [55]. While our study observed elevated suPAR in Group 1 and 4 PH, Butt et al. found no association between suPAR and PAH presence in systemic sclerosis, despite correlations with lung fibrosis markers [55]. This discrepancy suggests three cautious interpretations: (1) suPAR may reflect distinct pathological processes in different PH etiologies (vascular remodeling in our cohort versus fibrosis in SSc); (2) our PH-specific cohort design may have detected subtler associations than mixed SSc populations; or (3) suPAR’s modest elevations in PH could represent secondary inflammation rather than primary pathogenesis. Importantly, both studies agree on suPAR associates with organ dysfunction (fibrosis or PH severity) but disagree on pulmonary vascular specificity, highlighting the need for a etiology-specific biomarker validation. The proteomic analysis by Yokokawa et al. demonstrated that PLAUR (the suPAR precursor) failed to enhance discrimination in a 51-protein inflammatory panel for idiopathic PAH, potentially challenging suPAR’s specificity as a PH biomarker [56]. This finding contrasts with our observed association between suPAR’s and PH’s presence, possibly reflecting differences in study design (targeted suPAR measurement vs. discovery proteomics) or the examined PH subtypes (mixed Group 1 and 4 vs. idiopathic PAH). Together, these studies suggest that suPAR’s utility may depend on both analytical methodology and clinical context, warranting further investigation into its optimal application across PH phenotypes.

While our study confirmed significantly elevated suPAR levels in PH patients compared to controls (73.14 vs. 65.52 pg/mL, *p* = 0.012), the absolute difference was modest—approximately 12% higher than controls—and markedly smaller than the eleven-fold elevation seen with NT-proBNP. This raises critical questions about suPAR’s clinical applicability as a standalone biomarker. The modest change suggests that suPAR lacks the dynamic range required for robust discrimination between PH and non-PH states, particularly in early-stage disease or borderline cases. However, this does not necessarily negate its utility. In cardiovascular and inflammatory diseases, suPAR often exhibits smaller absolute differences than traditional biomarkers (e.g., CRP or troponins) but retains prognostic value due to its pathophysiological specificity for immune activation and endothelial dysfunction [14,19]. For PH, where inflammation and remodeling are central but heterogeneous drivers, suPAR’s modest elevation may reflect its role as a “bystander” marker of upstream pathways (e.g., TGF-β signaling or macrophage activation) rather than a direct mediator of hemodynamic stress (unlike NT-proBNP, which correlates tightly with ventricular strain) [53]. This could explain its stronger correlations with functional capacity (6-MWD) and ECM remodeling (Fibulin-1) than with pressure overload alone [51,52]. Clinically, suPAR’s utility may lie in composite models rather than isolated measurements. For example, integrating suPAR with NT-proBNP (to capture both inflammation and hemodynamic stress) or ECM markers (e.g., MMP-9) could improve risk stratification, particularly in inflammatory PH subsets (e.g., systemic sclerosis-associated PAH) [54,55]. Similarly, serial suPAR measurements might track treatment response to anti-inflammatory therapies (e.g., statins or immunomodulators) that do not alter NT-proBNP. Importantly, the lack of a “threshold effect” (i.e., no clear cut-off for PH diagnosis) mirrors challenges seen with other inflammatory biomarkers (e.g., IL-6) and underscores the need for population-specific validation to define clinically meaningful ranges. Future studies should assess whether suPAR’s modest elevation reflects dose-dependent relationships with outcomes (e.g., nonlinear mortality risk at higher levels) or identifies PH subtypes with distinct inflammatory profiles. Until then, suPAR’s role may be adjunctive—a “fine-tuning” tool for intermediate-risk cases or a research marker for mechanistic studies—rather than a primary diagnostic or prognostic test. The modest elevation of suPAR in our cohort prompts consideration of its variable utility across PH subgroups. In CTD-PH, suPAR may have a particular value given its strong association with both vascular injury and fibrotic progression. Studies demonstrate that suPAR correlates with pulmonary involvement in systemic sclerosis and tracks microvascular damage in CTD, suggesting that it could serve as a composite biomarker of disease activity in autoimmune- and autoinflammatory-driven PHs [54,55]. Conversely, in hypoxia-induced PH (Group 3), suPAR’s role appears more limited, as hypoxic vasoconstriction occurs largely independently of the inflammatory and fibrotic pathways that suPAR reflects. The interpretation of suPAR in PH is complicated by its nonspecific nature and sensitivity to comorbid conditions. Chronic heart failure (frequent in Group 2 PH) can elevate suPAR independently of pulmonary vascular disease, potentially explaining the 2-fold increase observed by Mirna et al. [57]. Similarly, renal dysfunction, metabolic syndrome, and chronic infections—all common in PH populations—may artificially inflate suPAR levels. This underscores the importance of (1) PH subgroup stratification when interpreting suPAR values and (2) comprehensive comorbidity assessment to avoid confounding. Future studies should employ multivariate models adjusting for these variables to isolate suPAR’s PH-specific signal.

In our study, univariate and multivariate logistic regression analysis showed that suPAR is independently associated with the presence of PH (*p* = 0.047 for univariate logistic regression analysis and *p* = 0.032 for multivariate logistic regression analysis, respectively, Table 2). On the other hand, suPAR levels correlated negatively with 6 min walk distance (6-MWD) (r= −0.310, *p* = 0.001) and tricuspid annular plane systolic excursion (TAPSE) (r = −0.295, *p* = 0.005) but positively with systolic pulmonary artery pressure (sPAP) (r = 0.241, *p* = 0.023) (Table 4). However, the observed correlations between suPAR and functional/hemodynamic parameters, while statistically significant, were modest in magnitude. Although these findings tentatively align with the hypothesis that higher suPAR levels may reflect poorer functional capacity and right ventricular dysfunction, the key prognostic factors and the weak effect sizes warrant caution in overinterpreting suPAR’s clinical utility [3,4,12]. Notably, the correlation with 6-MWD, though statistically significant, explains only ~9.6% of the variance (r^2^ = 0.096), suggesting that suPAR captures a limited aspect of functional impairment. Similarly, the association with TAPSE, while biologically plausible given suPAR’s putative role in ventricular remodeling, requires validation in larger cohorts to determine its clinical relevance [14]. Multiple linear regression confirmed 6-MWD as the sole independent predictor of suPAR levels (Table 5), further emphasizing that suPAR’s relationship with functional status, though measurable, is not robust enough to serve as a standalone severity marker. These results imply that while suPAR may contribute to PH pathophysiology, its weak correlations with established prognostic parameters highlight the need for larger studies to confirm these associations and for exploring suPAR within multimodal models (e.g., combined with NT-proBNP or imaging markers) to enhance its predictive value.

Interestingly, suPAR levels did not differ significantly between Group 1 and Group 4 PH (70.57 [63.90–235.76] pg/mL vs. 75.48 [62.31–80.08] pg/mL, *p* = 0.800), suggesting that suPAR may be a general marker of PH rather than specific to its etiology. This finding aligns with studies that indicate that suPAR reflects systemic inflammation and endothelial dysfunction, which are common features across PH subgroups. The lack of significant differences in echocardiographic and right heart catheterization parameters between the subgroups further supports this interpretation. To the best of our knowledge, our study is the first in the literature comparing patients with Group 1 and Group 4 PH in terms of suPAR levels.

There is one study in the literature investigating the suPAR levels in patients with PH. However, this study was designed not only for the assessment of suPAR levels but also for the analysis of five new-generation biomarkers, including suPAR, in patients with PH. Mirna et al. investigated the levels of sST2, H-FABP, GDF-15, and suPAR in patients with PH [57]. The main difference between this study and ours was the inclusion of all subgroups of patients with PH in this study. We did not include Group-2, Group-3, and Group-5 patients with PH. Similarly to our results, they demonstrated that serum levels of suPAR were significantly elevated in patients with PH when compared to controls and that the suPAR levels predicted the presence of PH [57]. The modest suPAR elevation (~12%) observed in our cohort contrasts with the 2-fold increase reported by Mirna et al., potentially reflecting differences in study populations—while we focused mainly on Group 1 and Group 4 PH, their inclusion of Group 2 PH (frequently complicated by systemic inflammation and heart failure comorbidities) may explain the more pronounced elevation. This discrepancy suggests that suPAR’s dynamic range varies by PH etiology, with greater increases likely occurring in phenotypes with stronger inflammatory components (e.g., left heart disease PH). Importantly, even our modest elevation remained independently associated with PH presence after adjustment, supporting suPAR’s potential role as a contributor to vascular pathology or a mirror of vascular pathology. Standardized multicenter studies comparing suPAR across PH subgroups are needed to clarify these etiology-dependent patterns and define clinically meaningful thresholds. Another important result of the study by Mirna et al. was that there was a statistically significant difference in suPAR levels among the PH subgroups. They showed that suPAR levels were significantly elevated in patients with Group 2 PH when compared to other subgroups of PH [57]. Although we did not see an assessment of whether there was a difference between Group-1 and Group-4 patients with PH in the study by Mirna et al., our results indicate that the suPAR levels were similar between Group-1 and Group-4 patients with PH (Table 3).

The elevation of suPAR levels in PH patients and its independent association with PH presence suggest that suPAR may serve as a valuable biomarker for risk stratification and disease monitoring. The correlation between suPAR and 6-MWD highlights its potential utility in assessing functional capacity, a critical endpoint in PH management. Furthermore, the inclusion of suPAR in multivariate models alongside established markers like NT-proBNP underscores its additive value in predicting PH. While our study observed no statistically significant difference in suPAR levels between Group-1 and Group-4 PH patients, several important limitations temper the interpretation of this finding. The small sample size of Group 4 (n = 8) and the heterogeneity of Group 1 (encompassing idiopathic PAH, CTD-PH, and other subtypes) may have limited our ability to detect potential subgroup-specific differences. These constraints suggest that the apparent lack of etiological specificity should be interpreted with caution, and larger, more homogeneous studies are needed to confirm whether suPAR truly reflects common pathways across PH subtypes. Similarly, while the elevated suPAR levels in PH patients could tentatively suggest a role for chronic inflammation in Group 1 and 4 PH pathophysiology, our study design did not include mechanistic investigations to confirm this relationship. Without direct measurements of inflammatory cytokines, cellular pathways, or experimental models, the contribution of inflammation remains speculative. Thus, while suPAR’s association with PH’s presence is notable, its pathophysiological significance—particularly as a marker of inflammation—requires further validation through targeted mechanistic studies. These limitations underscore that our findings should be viewed as preliminary. Future research with larger cohorts, stratified PH subgroups, and integrated mechanistic data will be essential to clarify suPAR’s role as a biomarker and its potential etiological specificity in PH.

### 4.1. Future Perspectives

Our study showed that suPAR levels are elevated in patients with Group 1 and Group 4 PHs. However, there is a lack of data in the literature about suPAR levels and PH, and we believe that further studies should be conducted to clarify the contribution of suPAR in the pathogenesis of PH. Future studies that may fill the gaps of this topic in the literature may be as follows.

- suPAR levels and long-term prognosis of patients with PH;
- The effects of PAH-specific treatments on suPAR levels;
- The effects of the inhibition of suPAR on the hemodynamic profile and long-term prognosis of PH;
- suPAR levels should be investigated in other subgroups of PH (Groups 2, 3, and 5);
- Large-scale multicenter studies are needed to clarify the cut-off suPAR levels, similar to NT-proBNP, for use in risk assessment and prediction of long-term mortality;
- After clarifying the cut-off values for suPAR in patients with PH, these values must be validated in an independent control group. On the other hand, investigating the effects of suPAR levels on the prediction power of the REVEAL 2.0 risk score should be valuable.

### 4.2. Study Limitations

Our investigation has some limitations. First, this is a single-center study, in which the number of patients recruited was relatively small. Second, this cross-sectional study was not designed to investigate the effects of suPAR levels on long-term prognosis. For this reason, we do not have any data about the association between suPAR levels and long-term prognosis of patients with PH. Despite this, in overall cohort, increased levels of suPAR remained independently associated with the presence of PH after multivariable analyses. Third, the measurements of suPAR levels were performed in patients who are currently under PAH-specific or targeted treatment. Therefore, our findings do not provide information about treatment-naive patients. Fourth, we could not show the levels of other inflammatory markers, such as IL-1, IL-6, and TNF-α, in this population. In addition, our study population comprised patients with Group 1 and Group 4 PHs, and our results cannot necessarily be applied to the other subgroups of PH.

## 5. Conclusions

Our study demonstrated that suPAR levels are significantly elevated in patients with group-1 and group-4 PH. This study indicates that low-grade, chronic inflammation may contribute to the pathogenesis of group 1 and Group 4 PH. More large-scale prospective studies are needed to clarify the role of suPAR as a biomarker and therapeutic target in patients with PAH and other forms of PH.

## Figures and Tables

**Table 1:** The baseline demographic, clinical, laboratory, and echocardiographic parameters of the study population.

	Control Group (n = 45)	PH Group (n = 44)	*p*
Group 1 PH, n (%)	-	36 (82)	
Group 4 PH, n (%)	-	8 (18)	
**Clinical parameters**			
Gender, male, n (%)	12 (26.7)	12 (27.3)	0.949
Age, years	51.24 ± 12.48	51.05 ± 18.18	0.952
BMI, kg/m^2^	27.38 ± 3.76	27.05 ± 3.73	0.674
Smoking, n (%)	3 (6.7)	2 (4.5)	1
Diabetes mellitus, n (%)	7 (15.6)	9 (20.5)	0.547
Hypertension, n (%)	11 (24.4)	11 (25)	0.952
Systolic blood pressure, mmHg	120 (100–130)	110 (100–130)	0.041 *
Diastolic blood pressure, mmHg	70 (70–70)	70 (62.50–80)	0.903
Heart Rate, beats per minute	70 (70–80)	82.50 (70–95)	0.005 *
6 min walking test, m	650 (622.50–680)	372.50 (312.50–407.50)	<0.001 *
**Laboratory parameters**			
WBC count, ×10^9^/L	7.72 ± 1.82	7.60 ± 2.92	0.802
Hemoglobin, g/dL	14.54 ± 1.41	12.65 ± 2.33	<0.001 *
Fasting blood glucose, mg/dL	94 (102–87.50)	93 (82.25–100.75)	0.290
GFR, mL/per minute	111.63 ± 19.48	103.71 ± 30.62	0.148
ESR, mm/h	13 (10.50–20.0)	18 (7.25–33.25)	0.160
CRP, mg/L	2.01 (1.05–3.03)	1.39 (0.60–3.56)	0.536
NT-proBNP, pg/mL	37.60 (24.40–80.50)	442.00 (120.75–1516.75)	<0.001 *
suPAR, pg/mL	65.52 (53.06–80.91)	73.14 (62.77–167.13)	0.012 *
**Echocardiographic parameters**			
LV end-diastolic diameter, mm	48 (46–49)	46(42–48.75)	0.024 *
LVEF, %	60 (60–60)	55.50 (55–60)	<0.001 *
Systolic PAB, mmHg	28 (26.50–29.50)	69 (53.50–80)	<0.001 *
TAPSE, mm	23 (21–24)	16 (14–18)	<0.001 *
RAA, cm^2^	15.00 (13.85–16.30)	27.55 (24.90–30.60)	<0.001 *
**Medications**			
RAS blockers, n (%)	11 (24.4)	17 (38.6)	0.149
Beta blockers, n (%)	13 (28.9)	13 (29.5)	0.946
Calcium channel blockers, n (%)	4 (8.9)	4 (9.1)	1
Statins, n (%)	4 (8.9)	8 (18.2)	0.230
Anticoagulant therapy, n (%)	1 (2.2)	16 (36.4)	<0.001 *
Oral anti-diabetics, n (%)	7 (15.9)	6 (13.6)	0.764

**Acronyms:** BMI, body mass index; CRP, C-Reactive Protein; ESR, erythrocyte sedimentation rate; GFR, glomerular filtration rate; LV, left ventricular; LVEF, left ventricular ejection fraction; NT-proBNP, N terminal pro brain natriuretic peptide; WBC, white blood cell; PAP, pulmonary artery pressure; PH, pulmonary hypertension; RAA, right atrial area; RAS, renin–angiotensin system; suPAR, soluble urokinase type plasminogen activator receptor; TAPSE, tricuspid annular plane systolic excursion. * a *p* value < 0.05 denotes statistical significance.

**Table 2:** Binary logistic regression analyses to determine independent associates of pulmonary hypertension presence.

Variables	Univariate		Multiple	
	OR (95% CI)	*p*	OR (95% CI)	*p*
Systolic blood pressure, mmHg	0.968 (0.940–0.998)	0.034	0.875 (0.780–0.981	0.022
Heart Rate, beats per minute	1.073 (1.027–1.121)	0.002	1.117 (0.951–1.314)	0.178
Hemoglobin, g/dL	0.583 (0.441–0.772)	<0.001	0.701 (0.266–1.848)	0.472
GFR	0.988 (0.971–1.005)	0.150	0.970 (0.899–1.047)	0.438
ESR, mm/h	1.043 (1.008–1.080)	0.016	1.177 (1.038–1.335)	0.011 *
NT- ProBNP, pg/mL	1.015 (1.007–1.024)	<0.001	1.025 (1.009–1.042)	0.003 *
suPAR, pg/mL	1.007 (1.016–1.032)	0.047	1.047 (1.004–1.093)	0.032 *
LV end-diastolic diameter, mm	0.896 (0.805–0.997)	0.044	0.877 (0.630–1.220)	0.435
LVEF, %	0.701 (0.581–0.846)	<0.001	0.901 (0.464–1.747)	0.757

**Acronyms:** CI, confidence interval; ESR, erythrocyte sedimentation rate; GFR, glomerular filtration rate; LV, left ventricular; LVEF, left ventricular ejection fraction; NT-proBNP, N terminal pro brain natriuretic peptide; OR, odds ratio; suPAR, soluble urokinase type plasminogen activator receptor. * a *p* value < 0.05 denotes statistical significance.

**Table 3:** The baseline demographic, clinical, laboratory, and heart catheterization parameters of the study population according to the type of pulmonary artery hypertension.

	Group 1 PH (n = 36)	Group 4 PH (n = 8)	*p*
**Clinical parameters**			
Gender, male, n (%)	9 (25)	3 (37.5)	0.663
Age, years	51.17 ± 19.19	50.50 ± 13.70	0.927
BMI, kg/m^2^	27.10 ± 3.89	26.85 ± 3.16	
Diabetes mellitus, n (%)	8 (22.2)	1 (12.5)	1.000
Hypertension, n (%)	11 (30.6)	0 (0)	0.170
6 min walking test, m	345.14 ± 71.07	437.50 ± 68.19	0.002 *
**Laboratory parameters**			
Hemoglobin, g/dL	12.72 ± 2.49	12.29 ± 1.50	0.637
NT-proBNP, pg/mL	520.50 (182.50–2516.75)	162.50 (69.72–585.75)	0.092
suPAR, pg/mL	70.57 (63.90–235.76)	75.48 (62.31–80.08)	0.800
**Echocardiographic parameters**			
LVEF, %	57 (55–60)	58 (55–60)	0.731
Systolic PAB, mmHg	74.11 ± 22.53	63.25 ± 25.26	0.234
TAPSE, mm	15.89 ± 2.20	16.87 ± 3.64	0.319
RAA, cm^2^	27.80 ± 3.77	27.95 ± 4.94	0.923
**Heart catheterization parameters**			
Systolic PAB, mmHg	77.42 ± 16.98	69.37 ± 24.37	0.270
Mean PAB, mmHg	46.97 ± 9.83	42 ± 14.21	0.241
Cardiac index, L/min/m^2^	2.48 ± 0.38	2.62 ± 0.49	0.383
PCWP, mmHg	10.75 ± 2.01	9.87 ± 0.35	0.230
Right atrial pressure, mmHg	9.44 ± 2.12	8.12 ± 2.23	0.122
PVR, Woods	7.62 ± 2.57	5.43 ± 4.29	0.063
**Medications**			
RAS blockers, n (%)	16 (44.4)	1 (12.5)	0.125
Calcium channel blockers, n (%)	4 (11.1)	0 (0)	1.000
Anticoagulant therapy, n (%)	11 (30.6)	8(100)	<0.001
**Pulmonary artery hypertension medications**			
Number of PH drugs			0.008 *
1, n (%)	14 (38.9)	8 (100)	
2, n (%)	16 (44.4)	0 (0)	
3, n (%)	6 (16.7)	0 (0)	
-Endothelin receptor antagonists			
Bosentan, n (%)	21 (58.3)	1 (12.5)	0.046 *
Macitentan, n (%)	12 (33.3)	0(0)	0.084 *
Ambricentan, n (%)	0 (0)	0 (0)	-
-Phosphodiesterase-5 inhibitors			
Sildenafil, n (%)	4 (11.1)	0(0)	0.434
Tadalafil, n (%)	18 (50)	0(0)	0.014 *
-Prostacyclin analogs			
Ilioprost (inhalated), n (%)	5 (13.99)	0 (0)	0.566
Epoprostenol (IV), n (%)	1 (2.8)	0 (0)	1
-Riociguat, n (%)	3 (8.3)	8 (100)	<0.001 *

**Acronyms:** BMI, body mass index; LVEF, left ventricular ejection fraction; NT-proBNP, N terminal pro brain natriuretic peptide; PAB, pulmonary artery pressure; PCWP, pulmonary capillary wedge pressure; PH, pulmonary artery hypertension; PVR, pulmonary vascular resistance; RAA, right atrium area; RAS, renin–angiotensin system; suPAR, soluble urokinase type plasminogen activator receptor; SVR, Systemic vascular resistance; TAPSE, tricuspid annular plane systolic excursion. * a *p* value < 0.05 denotes statistical significance.

**Table 4:** Correlation analyses between suPAR and baseline characteristics in study population.

	Study Population (n = 89) r Value	*p* Value
**Clinical parameters**		
Age, years	−0.055	0.644
BMI, kg/m^2^	0.124	0.247
Systolic blood pressure, mmHg	−0.030	0.777
Diastolic blood pressure, mmHg	0.030	0.783
Heart Rate, beats per minute	0.144	0.178
6 min walking test, m	−0.310	0.003 *
**Laboratory parameters**		
WBC count, ×10^9^/L	0.041	0.705
Hemoglobin, g/dL	−0.040	0.707
Fasting blood glucose, mg/dL	−0.065	0.545
GFR, mL/per minute	−0.047	0.659
ESR, mm/h	−0.010	0.990
C-reactive protein, mg/L	0.043	0.686
NT-proBNP, pg/mL	0.287	0.006
**Echocardiographic parameters**		
LV end-diastolic diameter, mm	0.034	0.752
LVEF, %	−0.016	0.885
Systolic PAB, mmHg	0.241	0.023 *
TAPSE, mm	−0.295	0.005 *
RAA, cm^2^	0.194	0.068
Cardiac Index, lt/m^2^	−0.047	0.764
PVR, woods	0.004	0.979
RAP, mmHg	0.133	0.390

**Acronyms:** BMI, body mass index; ESR, erythrocyte sedimentation rate; GFR, glomerular filtration rate; LV, left ventricular; LVEF, left ventricular ejection fraction; NT-proBNP, N terminal pro brain natriuretic peptide; WBC, white blood cell; PAB, pulmonary artery pressure; PVR, pulmonary vascular resistance; RAA, right atrial area; RAP, right atrial pressure; suPAR, soluble urokinase type plasminogen activator receptor; TAPSE, tricuspid annular plane systolic excursion. * a *p* value < 0.05 denotes statistical significance.

**Table 5:** Linear regression analyses for identifying independent associates of serum suPAR levels in the study population (n = 89).

Variables	Univariate			Multivariate		
	B ± SE	95% CI	*p* Value	B ± SE	95% CI	*p* Value
Heart Rate, beats per minute	3.012 ± 1.475	0.079–5.944	0.044	1.473 ± 1.591	−1.690–4.637	0.357
6-MWD, m	−0.363 ± 0.123	−0.607–(-)0.118	0.004	−0.363 ± 0.123	−0.607–(-)0.118	0.004 *
NT-proBNP, pg/mL	0.012 ± 0.016	−0.020–0.044	0.472	-	-	-
Systolic PAB, mmHg	1.316 ± 0.714	−0.103–2.735	0.069	−0.795 ± 1.104	−2.950–1.441	0.496
TAPSE, mm	−12.303 ± 4.873	−21.988–(-)2.619	0.013	−8.007 ± 9.217	−26.336–10.323	0.388
RAA, cm^2^	6.957 ± 2.751	1.489–12.424	0.013	0.515 ± 7.132	−13.669–14.700	0.943

**Acronyms:** NT- ProBNP, N terminal pro brain natriuretic peptide; PAB, pulmonary artery pressure; PHT, pulmonary artery hypertension; RAA, right atrial area; suPAR, soluble urokinase type plasminogen activator receptor; TAPSE, tricuspid annular plane systolic excursion; 6-MWD, six-minute walking distance. * a *p* value < 0.05 denotes statistical significance.

## Data Availability

The original contributions presented in this study are included in the article. Further inquiries can be directed to the corresponding author.
